# Video lecturing in Clicker-assisted English flipped class

**DOI:** 10.1371/journal.pone.0224209

**Published:** 2019-10-22

**Authors:** Yu Zhonggen

**Affiliations:** Department of English Studies, Faculty of Foreign Studies, Beijing Language and Culture University, Beijing, China; Fondazione Ugo Bordoni, ITALY

## Abstract

**Value:**

The study on the impact of video lecturing on clicker-assisted English flipped class was necessary because it has seldom been explored.

**Purpose:**

This study studied the impact of video lecturing on student satisfaction and English proficiency, plus correlations between student satisfaction levels and English proficiency.

**Methodology:**

Randomly recruited Chinese participants (Female N = 44; Male N = 43) from a university in China received both pre and post College English Test Band 4 and satisfaction measurements, together with a semi-structured interview.

**Findings:**

We concluded that the video-assisted class could cause significantly higher English proficiency than the non-video-assisted class (F = 23.17, *p <* .*001*, Partialη^2^ = .216); there were significant differences between video- and non-video-assisted cohorts for post interaction (F = 8.37, p = .005, Partialη^2^ = .093), post efficacy (F = 7.68, p = .007, Partialη^2^ = .086), and post regulation (F = 16.34, *p <* .*001*, Partialη^2^ = .166); there were strong, positive relationships between post English proficiency and post student interaction (R^2^ = .70; β = .84; p < .01), self-efficacy (R^2^ = .57; β = .75; p < .01) and self-regulation (R^2^ = .59; β = .77; p < .01) levels in both cohorts at the .*05* level. However, no strong, positive correlations were found in both cohorts at the .*05* level between pre English proficiency and pre student interaction (R^2^ = .00; β = .05; p = .33), self-efficacy [R^2^ = .03;β = -.17 (negative); p = .05] and self-regulation [R^2^ = .05;β = -.23 (negative); p = .01] levels. Future research into video-assisted English flipped class may need interdisciplinary cooperation.

## Introduction

The flipped or flipped pedagogical approach is a method which flips the teaching and learning process[1–2]. Through the flipped approach, participants were able to learn course contents at home first, thereby preparing themselves to participate in relevant learning activities, raise questions and solve problems with peers[3]. Online learning, in need of information technologies, accounts for a major component in the flipped approach since students are required to conduct self-directed learning outside class.

Clicker system, also referred to as classroom response system, is a popular information technology mainly composed of remote controllers, hand-held emitters, receivers, computers, multimedia projectors, screens and software. Using this system, students can anonymously poll and join learning activities involving peer discussion and problem solving, by which learning efficiency can be improved[4] and learning outcomes can be promoted[5]. Clickers enabled teachers to integrate learning activities into large-scale classrooms, where students’ attention was caught and prompt feedback was provided by the teachers to elucidate misunderstandings[6]. This study sheds light on the impact of videos on the flipped English (referring to English as a foreign language) class assisted with clickers.

Assisted with video lecturing designed and provided by teachers, the concepts hard to be perceived through texts, and graphics or PowerPoint slides may be easily understood[7]. Before class, the participants watched videos related to the topic of each lesson as one of the flipped strategies, which significantly increased collaborative peer dialogue among students[8]. However, it is also argued that the simple media fails to play an important role in academic achievements although academic contents can conveniently be delivered through the media to students[9]. Video-assisted tutorials increased motivation and performance and had significantly better results for training time, self-efficacy and scores in an immediate post-test[10] although the video-assisted online learning was reported more time-consuming than the traditional physical approach (Cavanaugh, 2005).

Considering the contradictory results, it is therefore important to determine the effectiveness of teaching tools, e.g. videos, slides, blackboard, multimedia projector, and online communicative technologies[7] in the clicker-assisted flipped English class. This study, aiming to identify the role of video lecturing in clicker-assisted flipped English class, is thus necessary.

Video-assisted learning was endowed with powerful features to constitute an effective pedagogical approach to improve academic achievements and student satisfaction[11]. With rapid development of online learning technologies, an increasing number of teachers recorded their video lecturing for students to watch in the form of mini-video, PowerPoint presentation, and Videocast[12]. Access to lectures is open to students through smart mobile phones, laptops, Wechat, YouTube or other technologies[13].

Videos provided students with convenience in English listening and speaking practice, together with easiness of relearning contents[14]. Videos-assisted students outperformed those who learned through traditional tools such as chalks and blackboard by providing them with plentiful opportunities of completing the missing contents[15].

Video lecturing also enabled students to understand previous lectures before they attended the physical or virtual class, facilitating their understanding and improving academic activities[16]. Video lecturing could present rich pictures and vivid voice for students to avoid confronting boring learning materials[17]. Significant differences in educational effectiveness were found between the instructions assisted with videos showing or hiding teachers’ faces[18].

Furthermore, in the context of online learning, video lecturing through images, sound, and other multiple inputs might enlarge capacity of working memory than the traditional blackboard and chalks-aided teaching[19]. They might also facilitate communication between students and learning textbooks by inspiring students[20].

Teachers and students were more interested in classes assisted with videos than in those without aid of videos[21]. The video lecturing, characteristic of mobile pictures, was indispensable to students who could conveniently and flexibly access teachers’ lectures through it (Fill and Ottewill 2006: 397). Assisted with video lecturing, students could choose to join lectures given by teachers and they could also play the video at the speed they desired[20], improving their learning efficiency.

Besides watching video lecturing, students could also discuss and finish the academic tasks assigned by teachers in the flipped classroom that involved blended learning[21]. This is beneficial to lecturing preparation and teaching practice[22].

The appropriate selection of pedagogy plays an important role in the asynchronous learning assisted with video lecturing. The video assisted pedagogy was beneficial to students’ academic activities[23]. Students could discuss difficult problems and answer either theoretical or practical questions in the flipped classroom[24].

As a popular teaching style, the flipped class integrated the asynchronous video lecturing into assignment, and cohort-based learning activities[25]. As a popular medium for learning outside class, videos are able to arouse student interest and stimulate student motivation, attract students and make learning resources easily accessible[26]. Although videos are typically used in the flipped pedagogy, they are not in use due to limited economic development and other factors in some areas. It is therefore necessary to determine the impact of video lecturing on the flipped pedagogy.

Furthermore, video lecturing-based pedagogical approach enjoyed students, reduced their anxiety and lessened their workload[27]. Video-assisted flipped class met students’ requirements in that students’ learning schedule could be freely arranged by themselves in the flipped approach although there were no statistically significant differences in satisfaction levels between the flipped and non-flipped approaches[28]. Assisted with learning technologies, e.g. smart mobile phones, learning platforms and other computer-based technologies, the flipped pedagogical approach made learning more flexible and convenient. Students held positive attitudes toward online learning technologies and they also reported that they could perceive academic contents better compared with the approach without aid of online learning tools[29].

In the flipped pedagogical approach, student participation in learning activities was encouraged and student satisfaction was also enhanced[30]. The videos as learning tools could positively influence learning process. Learning effect was thus improved if assisted with videos that were carefully designed because the properly designed videos could increase interactions between students and learning materials[31]. The video lecturing-assisted flipped classroom, different from the traditional teacher-centered approach, highlighted the student-centered teaching and learning method and improved interactions between peers and teachers[32].

However, video lecturing did not prove the best medium of instruction for introductory political science course. In the non-video-assisted course, the teacher and the course were more highly evaluated and students scored higher on exams than the video-assisted course[33], and there was no statistically significant difference in academic achievements and satisfaction between video and non-video assisted pedagogical approaches[31].

Despite that use of videos has been widely studied in the flipped class, the results were inconsistent and the effect of videos on clicker-assisted flipped English class has seldom been explored. Considering the research status quo, research questions are thus raised as: (1) Could the video-clicker-assisted flipped English class cause significantly better academic achievements than the non-video-clicker-assisted flipped English class? (2) Could the video-clicker-assisted flipped English class cause significantly higher student interaction, self-efficacy and self-regulation levels than the non-video-clicker-assisted flipped English one? (3) Are the post interaction, self-efficacy and self-regulation levels positively correlated with the post English proficiency in both pedagogical approaches? In order to answer this question, the pre interaction, self-efficacy and self-regulation levels (pretests) should be equivalent in both cohorts, and so should the pre-CET 4 results (pretests)[32].

Most literature supported the effectiveness of videos in flipped classes in terms of academic achievements and satisfaction. It has also been largely demonstrated that there is a positive relationship between academic achievements and satisfaction. Based on previous findings, three null hypotheses are raised as follows:

H1: The video-clicker-assisted flipped English class could not lead to significantly higher English proficiency than the non-video-clicker-assisted flipped English class.
H2: The video-clicker-assisted flipped English class could not cause significantly higher student interaction, self-efficacy and self-regulation levels than the non-video-clicker-assisted flipped English class.
H3: The post interaction, self-efficacy and self-regulation levels are not positively correlated with the post English proficiency in both pedagogical approaches. We assume that the pre interaction, self-efficacy and self-regulation levels are positively correlated with the pre-English proficiency in both pedagogical approaches.

## Methods

The research lacks consent because the data were analyzed anonymously. The research has been approved by the authors’ institutional review board-School of Foreign Languages of Hohai University, which waived the need for written informed consent from the participants. All experiments conform to the relevant regulatory standards.

The DOI link: http://dx.doi.org/10.17504/protocols.io.7sjhncn

This research attempts to integrate quantitative into qualitative research methods using several scales to measure English proficiency and satisfaction levels, together with a semi-structured interview to collect qualitative data. English was the linguistic medium of administration of questionnaires and interviews.

### Participants

Randomly selected 87 Chinese undergraduates (Male N = 43, Female N = 44) from a public university in China were willing to join the research, whose formal English education endured for approximately eight to nine years. They were normally literate and were in a normal psychological state based on their self-reports. They ranged from 19 to 22 in age (M = 20.33, S.D. = 1.02). They were assigned to two cohorts on a random basis, who received English teaching in the video-clicker-assisted flipped English class (N = 41) and the non-video-clicker-assisted flipped English class (N = 46) respectively. Both cohorts, under the instruction of the same teacher, had similar English proficiency identified through College English Test Band Four (CET 4). Both cohorts were assumed to be equivalent to each other in terms of pre-interaction, self-efficacy and self-regulation levels because they were randomly divided into two groups from the same population who shared the same educational and social backgrounds. This assumed similar English proficiency and interaction, self-efficacy and self-regulation levels established a basis for comparison.

Pre English proficiency and pre interaction, self-efficacy and self-regulation levels of the randomly selected participants were then determined via CET 4 (June, 2012) and the interaction, self-efficacy and self-regulation scales after they were randomly assigned to either intervention or control cohorts.

The teacher was awarded a Ph.D. degree in English language. Before the study commenced, he also received specialized training regarding both video-assisted and non-video-assisted pedagogical approaches.

Participants in the non-video-assisted cohort were asked to read textbooks before class without access to videos, while those in the video-assisted cohort had access to videos plus textbooks when preparing for class. The video-assisted cohort was required to complete all of the tasks that the non-video-assisted cohort completed and vice versa. Both cohorts of participants were instructed via clickers. The only difference was whether students could learn English assisted with videos. In the video-clicker-assisted flipped English class, students learned English aided with videos, while in the non-video-clicker-assisted flipped English class, they learned English without aid of videos.

### Research instruments

Four scales, including a CET 4 to determine English proficiency and three scales to identify interaction feasibility, self-efficacy and self-regulation levels, were used as research instruments in both the video-clicker-assisted flipped English class and the non-video-clicker-assisted flipped English class.

#### CET 4

CET 4 in June, 2012, designed under the guideline of Ministry of Education of China, was used to identify English proficiency. CET4 was internally reliable and externally valid. Its identification of English proficiency has been generally accepted since it came into being decades ago[33]. Test items in the CET 4 include writing, listening comprehension, reading comprehension, and translation (See Table 1).

**Table 1:** Details of CET 4.

Structure	Content	Percentage	Duration
Writing	Writing based on requirements	15	30m
Listening comprehension	Dialogue	15	30m
	Paragraph	20	
Reading comprehension	Lexical understanding	5	40m
	Skimming and scanning	10	
	Detailed reading	20	
Translation	From Chinese to English	15	30m
Total		100	130m

As shown in Table 1, there are in total four sections in the CET 4. Section 1 requires test takers to complete writing a short essay within 30 minutes. They should write at least 120 words following the given outline. Section 2 includes three parts: 8 short conversations and 2 long conversations, 3 short passages, and a passage. Section 3 is made of three parts: skimming and scanning, blank filling in a passage, and in-depth reading comprehension. Section 4 requires test takers to complete the sentences by translating into English the Chinese given in brackets.

#### The scales to identify interaction feasibility, self-efficacy and self-regulation levels

Interaction, self-efficacy and self-regulation were three key factors influencing student satisfaction[32]. Peer and student-teacher interactions, as well as interaction between students and learning contents could also play an important role in student satisfaction[34]. In the environment of clicker-assisted education, student self-efficacy exerted an important influence on student satisfaction (Liang and Tsai, 2008; Artino and Anthony, 2007). As regards to youngsters, student self-efficacy was closely related to satisfaction (Çakar, 2012). Self-regulation was referred to as the variable revealing student meta-cognition and motivation (Zimmerman and Schunk, 1989), which exerted a positive influence on student satisfaction (Deci and Ryan, 1996; Bembenutty and White, 2013). Three scales, followed by a five-point Likert scale: *I strongly disagree*, *I disagree*, *neutral*, *I agree*, *I strongly agree*, were used to identify interaction feasibility, self-efficacy and self-regulation respectively (see Table 2).

**Table 2:** A scale to identify satisfaction[32].

Scales of satisfaction	α
**Interaction feasibility**	.82
Clickers-assisted English class provides a discussion platform for interaction.	
Clickers-assisted English class facilitates feedback from peers.	
Clickers-assisted English class presents an easy access to frequently asked questions.	
Clickers-assisted English class provides a place to discuss questions.	
Generally, Clickers-assisted English class contributes to the interactive capacity of students.	
**A self-efficacy scale**	.90
I feel confident understanding terms or words relating to hardware of clickers.	
I feel confident understanding terms or words relating to software of clickers.	
I feel confident describing functions of clickers.	
I feel confident trouble shooting problems of clickers.	
I feel confident explaining why a task will not run through clickers.	
I feel confident using the clickers to learn.	
I feel confident learning advanced skills through clickers.	
I feel confident turning to a peer discussion when needed.	
**A self-regulation scale**	.83
If I study in appropriate ways, then I will be able to learn the material in this course.	
It is my own fault if I don't learn the material in this course.	
If I try hard enough, then I will understand the course material.	
If I don't understand the course material, it is because I didn't try hard enough.	

**Likert scale:** 5 = strongly agree; 4 = agree; 3 = neutral, 2 = disagree; 1 = strongly disagree

As shown in Table 2, questions to identify interaction centered on student-student and student-teacher interaction since both interactions were important indicators of satisfaction[34]. Sample questions are: “Clickers-assisted English class presents an easy access to frequently asked questions; Clickers-assisted English class provides a place to discuss questions.” Self-efficacy mainly referred to student beliefs, confidence, and expectations in accomplishing tasks[35]. Therefore, the scale to identify self-efficacy focused on these three elements. Examples are “I feel confident explaining why a task will not run through clickers; I feel confident using the clickers to learn; I feel confident learning advanced skills through clickers; I feel confident turning to a peer discussion when needed.” Self-regulation indicated the extent that students meta-cognitively, motivationally, and behaviorally fulfilled learning tasks[36]. Therefore, questions aiming to identify self-regulation, as shown in Table 2, pivoted on student meta-cognition, motivation and behaviors. Sample questions are: “If I try hard enough, then I will understand the course material; If I don't understand the course material, it is because I didn't try hard enough.”

Although there is not a specific item evaluating student–teacher interaction in the interaction scale, the following three questions do identify student-teacher interaction: (1) Clickers-assisted English class provides a discussion platform for interaction; (2) Clickers-assisted English class presents an easy access to frequently asked questions; (3) Clickers-assisted English class provides a place to discuss questions.

Cronbach’s alpha is a method to examine reliability, which was proposed by Lee Cronbach in 1951. It overcomes the shortcomings of partial halving method and is the most commonly used reliability analysis method in social science research. In general exploratory studies, the Cranbach’s alpha coefficient is above 0.6, and the benchmark study is above 0.8[37].

The internal consistency was demonstrated by Cronbach’s alphas (see Table 2). Three sub-scales to determine interaction feasibility, self-efficacy and self-regulation were all considered internally consistent since they reached the satisfactory level (α > .80) in the study.

#### A semi-structured interview

A semi-structured interview, designed by professors in English language teaching, was designed in order to collect qualitative data. Researchers informed participants that all the information in the interview would remain confidential and would merely be used in the research. All the interviewees would be properly rewarded for their cooperation after the interview.

The interview is made of three sections involving demographic information, interview questions and acknowledgment. Section 1 focuses on interviewees’ demographic information including names, ages, genders, educational backgrounds, working experiences, literacy and psychology. Section 2 is mainly concerned with interview questions. For the interaction feasibility, we selected 2 to 3 questions for interviewees to answer. For the scale of self-efficacy, 3 to 4 questions were selected. For the scale of self-regulation, we selected 2 to 3 questions (See Table 2). We also designed some questions regarding English listening, speaking, reading, writing and translation skills in order to solicit the data of interviewees’ English proficiency. The last section aims to extend our gratitude to all the interviewees and their contribution to the study.

### Research procedure

Learning tasks of participants were to improve their English proficiency through an intensive English reading course for two semesters. Both cohorts learned English assisted with Clicker system.

#### The teaching process assisted with Clicker system

**Fig 1:**
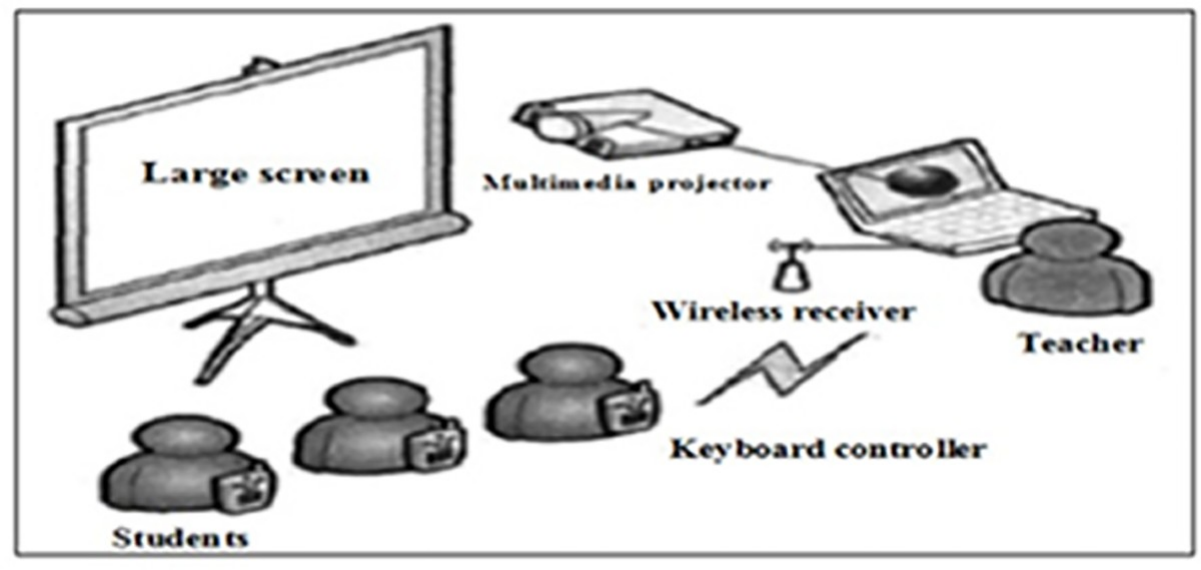
The teaching process assisted with Clicker system.

The teaching progress assisted with Clicker system is visualized in Fig 1.

As shown in Fig 1, assisted with clicker system, the teacher could project language notes to the large screen in order to present the lecture and ask students to follow. The teacher could also raise questions for students to answer. Using keyboard controller, students could answer questions, which was transmitted through the wireless receiver to the teacher. The teacher could then show students’ answers on the large screen, which would be displayed in the form of histograms. The teacher and students could easily judge the answers.

The teaching progress was decided based on the answers of students. If most of students (>80%) answered correctly, the teacher would move on after correcting the wrong answers. If nearly half students answered correctly, the teacher would stop to explain the question in details and continue the teaching progress after they understood them. If most of the students answered wrongly, the teacher would repeat the teaching progress after most of them learned the knowledge, understood the questions and provided correct answers.

#### The teaching process for both cohorts

The intervention cohort was instructed through the video-clicker-assisted flipped English class, while the control cohort was taught via the non-video-clicker-assisted flipped English class instruction.

The video was used to both present the image of the teacher and show educational materials. The intervention cohort was provided with teaching videos through the communicative platform. They were required to download videos from the online space. The activities were automatically recorded by the platform. In both the physical and virtual classes, the teacher designed quizzes or tests in the content of the videos in order to encourage students to view and understand videos. Task and key knowledge were included in the videos. Students could not successfully perceive the knowledge and complete the task without watching videos, which ensured that the intervention cohort truly made use of the videos.

After two academic semesters, both cohorts were tested in terms of English proficiency and interaction, self-efficacy and self-regulation levels. The pre and post test results were entered into a computer for further analysis and discussion. The only difference between the intervention cohort and the control cohort was that the former was instructed assisted with videos, while the latter was not instructed with the aid of videos. Consequently, differences in student interaction, self-efficacy and self-regulation levels and English proficiency were due to whether video lecturing was used. The effect of videos on clicker-assisted flipped English class would thus be revealed.

The videos, enduring 10 to 50 minutes, were all designed by the teacher, who aimed to inspire and encourage students to improve their listening, speaking, reading, writing and translating skills. Cultural awareness and linguistics-related knowledge would also be integrated into language teaching via the videos.

## Results

This section includes the results of the assumption and hypotheses, together with those from the semi-structured interview.

### Tests of the assumption

In order to test the hypotheses using an appropriate statistical test, the pre interaction, self-efficacy and self-regulation levels and pre English proficiency were assumed equivalent in both cohorts.

A one-way between-subjects ANOVA was conducted to compare the effect of video or non-video assisted conditions on pre interaction, self-efficacy and self-regulation levels and pre English proficiency. There was insignificant effect of pre interaction (*F =* .*739*, *p =* .*392*), self-efficacy (*F =* .*202*, *p =* .*654*) and self-regulation levels (*F =* .*073*, *p =* .*788*) and pre English proficiency (*F =* .*529*, *p =* .*469*) at the .*05* significance level for video or non-video assisted conditions. Taken together, pre interaction, self-efficacy and self-regulation levels and pre English proficiency are not significantly different in video or non-video assisted conditions. Our results suggest that the pre interaction, self-efficacy and self-regulation levels and pre English proficiency are equivalent in both cohorts.

### Tests of hypotheses

To test Hypothesis 1, we considered video or non-video lecturing condition as a fixed factor, and post English proficiency as a dependent variable (see Table 3).

**Table 3:** Tests of between-subjects effects with regard to english proficiency.

Dependent Variable:Post-CET 4						
Source	Type III Sum of Squares	df	Mean Square	F	Sig.	Partial Eta Squared
Corrected Model	15733.449^a^	2	7866.724	352.57	.000	.894
Intercept	365.782	1	365.782	16.39	.000	.163
Pre-CET 4	14686.132	1	14686.132	658.21	.000	.887
video	516.902	1	516.902	23.17	.000	.216
Error	1874.229	84	22.312			
Total	2.208E7	87				
Corrected Total	17607.678	86				

^a.^ R Squared = .894 (Adjusted R Squared = .891)

As shown in Table 3, a one-way ANCOVA was conducted to compare the effect of video and non-video lecturing whilst controlling for pre English proficiency. Levene’s test and normality checks were carried out and the assumptions met. There was a significant difference in mean post English proficiency (*F = 23*.*17*, *p<0*.*001*, *Partial*η^*2*^ *=* .*216*) between video and non-video lecturing-assisted cohorts. Post hoc tests showed there was a significant difference between video lecture and non-video lecture assisted cohorts (*p<0*.*001*). Comparing the estimated marginal means showed that the video lecturing-assisted cohort (*M = 5*.*058*) obtained significantly higher English proficiency than the non-video lecturing-assisted cohort *(M = 5*.*009*). Therefore, we rejected the null hypotheses “the video-clicker-assisted inverted English class could not lead to significantly higher English proficiency than the non-video-clicker-assisted inverted English class”.

To test Hypothesis 2, we included video or non-video lecturing condition as a fixed factor, and post satisfaction levels (interaction, self-efficacy and self-regulation) as dependent variables (see Table 4).

**Table 4:** Tests of between-subjects effects with regard to satisfaction levels.

Source	Dependent Variable	Type III Sum of Squares	df	Mean Square	F	Sig.	Partial Eta Squared
Corrected Model	postinteraction	72.785^a^	4	18.196	2.56	.044	.111
	postefficacy	241.864^b^	4	60.466	3.02	.022	.128
	postregulation	79.256^c^	4	19.814	4.90	.001	.193
Intercept	postinteraction	117.371	1	117.371	16.54	.000	.168
	postefficacy	464.542	1	464.542	23.22	.000	.221
	postregulation	88.440	1	88.440	21.86	.000	.211
video	postinteraction	59.381	1	59.381	8.37	.005	.093
	postefficacy	153.695	1	153.695	7.68	.007	.086
	postregulation	66.085	1	66.085	16.34	.000	.166
Error	postinteraction	581.943	82	7.097			
	postefficacy	1640.578	82	20.007			
	postregulation	331.695	82	4.045			
Total	postinteraction	23924.002	87				
	postefficacy	60876.582	87				
	postregulation	17215.152	87				
Corrected Total	postinteraction	654.728	86				
	postefficacy	1882.441	86				
	postregulation	410.951	86				

a. R Squared = .111 (Adjusted R Squared = .068)

b. R Squared = .128 (Adjusted R Squared = .086)

c. R Squared = .193 (Adjusted R Squared = .153)

As shown in Table 4, a series of multivariate ANCOVAs were used to compare the satisfaction levels between the video and non-video assisted instructions, with post interaction, post self-efficacy and post self-regulation levels as dependent variables, video or non-video lecturing conditions as fixed factors, and pre interaction, pre self-efficacy and pre self-regulation levels as covariates. The multivariate result was significant for use of video lecturing, *Pillai’s Trace =* .*169*, *F = 5*.*41*, *df = 3*, *p =* .*002*, indicating a significant difference in the satisfaction levels of student use of video lecturing between both cohorts. The univariate *F* tests showed there were significant differences between video lecturing-assisted and non-video lecturing-assisted cohorts for post interaction (*F = 8*.*37*, *df = 1*, *p =* .*005*, *Partial*η^*2*^ *=* .*093*), post efficacy (*F = 7*.*68*, *df = 1*, *p =* .*007*, *Partial*η^*2*^ *=* .*086*), and post regulation (*F = 16*.*34*, *df = 1*, *p <* .*001*, *Partial*η^*2*^ *=* .*166*) with respect to satisfaction levels. Therefore, we rejected the null hypotheses “the video-clicker-assisted inverted English class could not cause significantly higher student satisfaction than the non-video-clicker-assisted inverted English class”.

In order to test the relationship between English proficiency and student interaction, self-efficacy and self-regulation levels, data collected from the corresponding scales were entered into WarpPLS 6.0 for analysis since WarpPLS is especially reliable for small samples[38]. A WarpPLS model was established where the correlation between pre English proficiency and pre student interaction, self-efficacy and self-regulation levels was formulated to establish a baseline for comparison between post English proficiency and post student satisfaction. Furthermore, the correlational model between post English proficiency and post student interaction, self-efficacy and self-regulation levels was also formulated to test the hypothesis.

When assessing the model fit with the data, several criteria need to be recommended. In this study, average path coefficient (APC) is 0.466, p < 0.001; the average R-squared (ARS) is 0.323, P < 0.001; average adjusted R-squared (AARS) is 0.315, P < 0.001. The model fit is thus recommended since the P values for the APC, ARS and AARS are all equal to or lower than 0.05[39]. Furthermore, in this study, average full collinearity VIF (AFVIF) is 4.131, which is acceptable since the acceptable VIF is equal to or less than 5[39]. Tenenhaus GoF (GoF) is 0.568, which is highly acceptable since explanatory power of a model is considered highly acceptable if GoF is equal to or larger than 0.36[39]; Sympson’s paradox ratio (SPR) is 1.000, which is perfect since it is ideally accepted if SPR is 1, so are R-squared contribution ratio (RSCR) and statistical suppression ratio (SSR)[39]. Finally, nonlinear bivariate causality direction ratio (NLBCDR) is 0.750, which is acceptable since it is considered acceptable if NLBCDR is equal to or larger than 0.7[39]. To sum up, the model fit and quality indices have reached an acceptable level on the basis of the suggestion[39].

**Fig 2:**
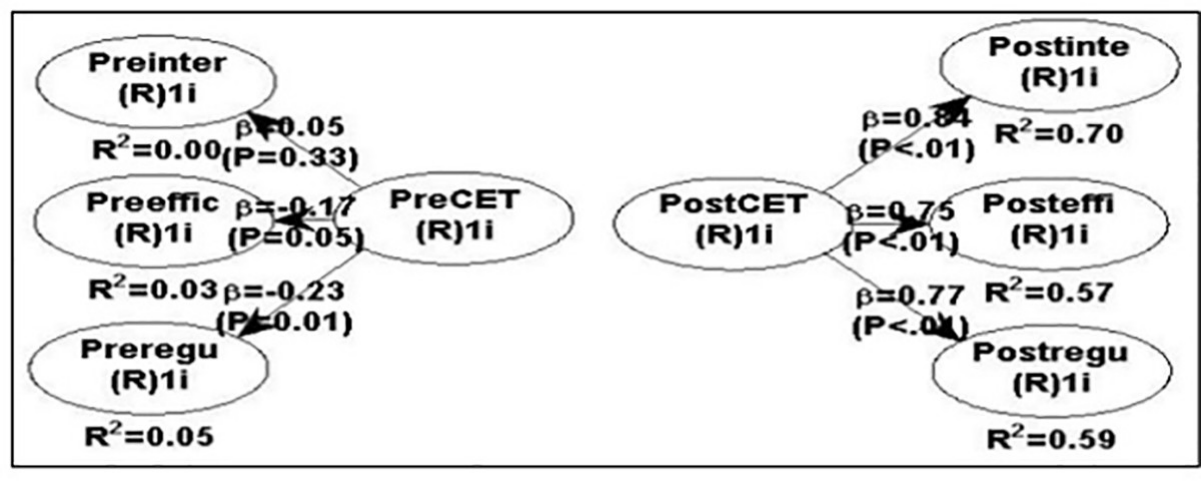
A WarpPLS model for correlational analysis. Notes: Pre/Post-CET4:Pre/Post College English Test Band 4;Pre/Postine(r):Pre/Post interaction;Pre/Posteffi(c):Pre/Post self-efficacy;Pre/Postregu:Pre/Post self-regulation.

Both pre and post analyses were conducted simultaneously through the correlational analysis of WarpPLS model (See Fig 2).

It can be found from Fig 2 that after two semesters’ treatment, there are strong, positive relationships between post English proficiency and post student interaction (*R*^*2*^ *=* .*70;* β *=* .*84; p <* .*01*), self-efficacy (*R*^*2*^ *=* .*57;* β *=* .*75; p <* .*01*) and self-regulation (*R*^*2*^ *=* .*59;* β *=* .*77; p <* .*01*) levels under both the video-clicker-assisted and non-video-clicker-assisted inverted English class models at the .*05* level. However, no strong, positive correlations between pre English proficiency and pre student interaction (*R*^*2*^ *=* .*00;* β *=* .*05; p =* .*33*), self-efficacy [*R*^*2*^ *=* .*03;* β *= -*.*17* (negative); *p =* .*05*] and self-regulation [*R*^*2*^ *=* .*05;* β *= -*.*23 (negative); p =* .*01*] levels were found in both video- and non-video-clicker-assisted cohorts at the .*05* level. As a result, we rejected the null hypothesis “the post interaction, self-efficacy and self-regulation levels are not positively correlated with the post English proficiency in both pedagogical approaches”, and the assumption “the pre interaction, self-efficacy and self-regulation levels are positively correlated with the pre English proficiency in both pedagogical approaches” was violated.

To enhance the reliability of the correlational analysis, a bivariate correlation program was conducted. The results are shown in Table 5.

**Table 5:** Correlation between student interaction, self-efficacy and self-regulation levels and english proficiency.

Correlated variables	Pearson correlation coefficient	P
Pre-CET 4- Preinteraction	.021	.845
Pre-CET 4- Preefficacy	-.039	.721
Pre-CET 4- Preregulation	-.027	.804
Post-CET4-Postinteraction	.743**	.000
Post-CET 4- Postefficacy	.656**	.000
Post-CET 4- Postregulation	.652**	.000

**. Correlation is significant at the 0.01 level (2-tailed).

Notes: Pre/Post-CET 4: Pre/post English proficiency.

Pearson correlation coefficients were computed to assess the relationship between student interaction, self-efficacy and self-regulation levels and English proficiency. As shown in Table 5, the pre English proficiency was not significantly correlated with the pre student interaction (*r =* .*021*, *p =* .*845*), self-efficacy (*r = -*.*039*, *p =* .*721*) and self-regulation (*r = -*.*027*, *p =* .*804*) levels, while there was a strong, positive correlation between the post English proficiency and the post student interaction (*r =* .*743*, *p <* .*001*), self-efficacy (*r =* .*656*, *p <* .*001*) and self-regulation (*r =* .*652*, *p <* .*001*) levels. The results are consistent with those obtained from the WarpPLS model and therefore enhance the reliabilities of rejecting the third null hypothesis and the assumption.

### Results from the interview

The interview was recorded and the recording was then transcribed for further analysis. The results are generally consistent with the quantitative research findings based on CET 4 and satisfaction scales. Specifically, the results were divided into two sections: opinions of interviewees and the information revealed.

#### Opinions of interviewees

We randomly selected 35 interviewees on a voluntary basis. Most of them felt that the video-clicker-assisted flipped English class could help them reach a higher English proficiency level than the non-video-clicker-assisted flipped English class. They reported that the video-clicker-assisted flipped English class was more satisfactory than the non-video-clicker-assisted flipped English class. It was believed that the post satisfaction might exert an important influence on the post English proficiency in either video or non-video assisted approach. They assumed that their pre satisfaction levels were similar and their satisfaction levels with the video and non-video assisted approaches were not significantly different before they joined the research.

Many participants thought that videos could expedite the pedagogical process since teachers do not need to painstakingly write with chalks on the blackboard. Instead, they could deliver knowledge assisted with educational technologies such as online recording, typing, and presenting. Participants said, “Videos light us up; They are time-saving; They energize us.” They could also share resources among students asynchronously. Videos including large quantities of contents could undoubtedly increase learning efficiency. Videos could also act as a tutor guiding students through the learning process. Many participants said, “Videos are highly efficient; Videos share information quickly and widely; Videos carry a large amount of information.”

It was reported by most interviewees that video lecturing provided them with flexibility, accessibility, and diversity when they achieved academic achievements by meeting various individual learning needs via pieces of knowledge and by accommodating individual needs via rich resources. Video lecturing was also available to students day and night, and students were able to watch video lectures from any internet-connected, video-play device such as smart phones and laptops. Interviewees thus believed that their learning styles were flexible with regard to their timetables, with which students with different learning styles were satisfied since they could flexibly choose the time and venue to watch or review lectures. Many interviewees thought that they could apply and appreciate the videos that enabled them to pause, rewind or replay at their will.

Note taking and note reviewing were essential learning activities, which was demonstrated by a tremendous number of studies[40]. Interviewees said that they could also obtain the opportunity to produce neatly-written and well-organized notes and achieve better academic achievements when allowed to watch the videos for a second time. Quality notes motivated them to review the notes, which could exert a beneficial influence on academic achievements. The process of note-taking itself could also enhance the acquisition and retention of knowledge, leading to higher English proficiency.

Nevertheless, interviewees also showed their worry about the possibility of increasing teachers’ cognitive load in that teachers had to spend a huge amount of time and energy in recording and designing the videos. Furthermore, the benefits of video lecturing failed to be demonstrated as better results in English intensive reading courses. They complained that they preferred to read the static books rather than read books when walking. They also sometimes considered video lecturing a way to distract them from reading due to the noise along with the video play. When forced to read the mobile materials, they tended to suffer from eye strain, which discouraged them from continuously watching the videos.

#### Information revealed

The qualitative accounts provided by the participants also revealed some important information. A phenomenon worth attention is participants’ report on their feelings about use of videos. For example, numerous students gave detailed accounts of experiencing pleasure and enjoyment about video viewing. They said, “I like watching videos; I enjoy watching videos; Learning through the video is interesting.” They also expressed their negative feelings about the lecturing without videos, e.g., “I hate reading the boring texts; I don’t like following the teacher; Listening to the teacher is sometimes boring.”

Besides, participants detailed their positive feelings about English learning with the assistance of videos. Many of them expressed similar feelings. Examples are “watching videos can improve my English listening skills; I can improve my sense of English language through viewing videos; Videos can improve my English pronunciation; Watching videos can improve my English skills.” By contrast, they disliked the English learning style without aid of videos. They said, “Learning without videos disgusts me; No lively pictures can be seen without videos; No videos, no English.”

## Discussion

This study arrived at the conclusion that the video-clicker-assisted flipped English class could lead to significantly higher levels of English proficiency and student interaction, self-efficacy and self-regulation than the non-video-clicker-assisted flipped English class; the post interaction, self-efficacy and self-regulation levels are positively correlated with the post English proficiency in the video or non video-assisted pedagogical approach; the pre interaction, self-efficacy and self-regulation levels are not significantly correlated with the pre English proficiency in both pedagogical approaches. The findings of this study are generally consistent with previous works[33, 41, 32, 42, 15].

Assisted with videos, students could freely learn the difficult contents they feel necessary. They may speed up, rewind or pause to meet their own needs. Videos made of interesting stories, vivid sounds and colorful pictures could have attracted student attention, due to which students might concentrate on the learning contents. In this way, their English proficiency could doubtlessly be improved. Pieces of knowledge in the videos could also be helpful for students to construct organized and systematic schema. It is therefore reasonable to find that video-assisted academic achievements are significantly higher than those without videos.

Interaction, self-efficacy and self-regulation constitute the elements that play key roles in student satisfaction[32]. To determine whether the video-clicker-assisted flipped English class is satisfactory, it is necessary to measure these three factors. Interactions between videos, online resources and students are facilitated when students watch videos and communicate with peers using online technologies. Communication with peers and teachers can undoubtedly improve student self-efficacy. Clicker-assisted performance checking encourages students to cultivate their self-regulation. Students could also be satisfied with the flexible learning style aided with technologies. Higher English proficiency in the video-assisted flipped English class could also have increased student satisfaction levels. It is therefore reasonable to conclude that the video-assisted flipped English class could improve student interaction, self-efficacy, and self-regulation.

The positive correlation between post English proficiency and post interaction, self-efficacy and self-regulation levels indicates that the higher English proficiency is, the more satisfied students will feel and vice versa. In the clicker-assisted flipped English class, student interaction, self-efficacy and self-regulation levels are important factors influencing post English proficiency. To improve student interaction, self-efficacy and self-regulation levels may be a decisive strategy to raise the English proficiency level in the clicker-assisted flipped English class whether or not videos are in use. Interaction, self-efficacy and self-regulation play important roles in student satisfaction. English proficiency is closely related with self-efficacy, self-regulation, peer interaction, and student-content interaction. Thus interaction, self-efficacy, and self-regulation are strongly correlated with English proficiency.

## Conclusions

This part aims to summarize the research in terms of advantages, disadvantages and future research directions.

### Advantages

Despite this study finds that video lecturing is positively correlated with student interaction, self-efficacy and self-regulation levels and academic achievements, no evidence of this finding has been found in previous empirical studies[43, 44]. Findings of this study were validated and enhanced through the integration of quantitative and qualitative research methods. The sample is large enough to represent the population. Cronbach’s alpha coefficients were computed to test the reliability of this study. Except for the ANCOVA used to analyze English proficiency and interaction, self-efficacy and self-regulation levels, WarpPLS model was also formulated to analyze the relationship between English proficiency and student interaction, self-efficacy and self-regulation levels. Nine global model fit and quality indices are provided, which establishes the reliability of the modelling analysis. Qualitative data collected from the interview strengthened the reliability of this study.

### Disadvantages

Although extensively explored, satisfaction is still an elusive variable that can hardly be fully measured. Measurement of satisfaction may need highly advanced and precise technologies. Questionnaires may never be perfect scales to identify satisfaction. The elements of satisfaction may not be limited to interaction, self-efficacy and self-regulation. Other elements may also be important factors related to satisfaction. Additionally, features of videos may vary from one to another. Features of video lecturing may be an inescapable factor in this study.

Many video lectures are simply videos of the face-to-face lecture and therefore, many of the advantages may not be applicable. There may be no rationale for the video lecturing design if interesting pictures and other engaging techniques can’t be presented in the classroom. Video lectures can be rewound or sped up, but they may fail to offer the possibility of clarification that can be realized through face-to-face pedagogy.

The double blind approach was not used since it was impossible that the researchers who interacted with the participants did not know which cohort was the intervention group and which was the control group.

### Future research directions

Students with various economic statuses may perform differently on the video lecturing-assisted teaching. Those who are better-off may have more convenient access to technologies and have better skills than those who are economically lower. The economic status may be considered an important factor in the research of educational technologies.

The Type of videos is also a topic worth exploring. As mentioned, rich moving pictures, vivid voice, connected plots, and background music may help students perceive the main contents and command the linguistic knowledge. Future research may thus focus on the effect of videos carrying different information in the flipped pedagogy. Measurement of psychology may be in need of advanced technologies and interdisciplinary cooperation. Interdisciplinary research involving psychology, education, statistics, linguistics and mathematics may be necessary in the future research.

## Supporting information

S1 Dataset file S1_dataset of video lecturing in CFLC.rar. (RAR)
